# Address Delivered before the Academy of Dental Science

**Published:** 1874-11

**Authors:** W. W. Allport

**Affiliations:** Chicago


### ADDRESS DELIVERED BEFORE THE AMERICAN ACADEMY OF DENTAL SCIENCE, AT ITS SEVENTH ANNUAL MEETING, HELD IN BOSTON, SEPT. 28th, 1874. PUBLISHED BY VOTE OF THE ACADEMY.

BY W. W. ALLPORT, CHICAGO.

*Mr. President and Fellows of the American Academy of Dental Science:*

In an annual address, on an occasion like this, it would seem that remarks bearing upon the interests of our profession at large, rather than upon any particular question, practice, or science would be appropriate.

The oft-recurring meetings of members of our profession, in different parts of our own country, as well as in Europe in which papers are presented evincing close observation and careful study upon modes of practice in the different departments of dental science; the multiplication of our text books and the well filled pages of our periodical literature, our dental colleges with many of their chairs filled with teachers qualified to instruct in medical colleges as well as the high professional character mantained by many private practitioners, are, I think, sufficient evidence that there is not only an earnest desire, but a *determination* on the part of the better members of our profession for advancement and a correct application of dental science.

That within the last thirty or forty years rapid and substantial progress has been made in this direction, has been fully demonstrated in the experience of some of the older members of this Academy, as well as by those of like age and experience throughout our country.

But because so much has been accomplished within this time or during the present century let us not be boastful; for, when we consider the changes and improvements that have been made in the science and practice of medicine, both general and special, in husbandry, in the arts, and in the sciences in general, there is not a little reason to question whether we in our particular calling, have, more than kept pace with the progress and developments in the other fields of science and labor around us.

Dentistry as a special art or department of science is, it is often said, mainly, at least, of modern origin. It is an outgrowth, partly, of an increased ratio of disease in the teeth, though not so much probably as it is of a new demand of the advanced civilization and culture of the age. At an early day —far back in the past—when education and refinement were less general, teeth, undoubtedly, suffered decay and gave trouble, but less being known of the causes of disease and less of correct treatment, and less attention being paid to personal appearance, less importance was attached to the teeth than at the present time. But, as civilization advanced and and refinement increased, health rose in estimation, cleanliness and personal appearance received more attention, and as a consequence, defective and unsightly teeth became objects of greater concern, and dentistry, at first rude, came up and has grown to its present magnitude and perfection, to meet this new demand of the times, just as the printing press the telegraph and steam locomotion were first brought into existence, with all their imperfections, and have by ingenuity and skill, from time to time, been improved to meet the demand created by the growth of business and desire for progress and information incident to the increase of population in our country and the spread of the civilization of the century.

When, therefore, we are persuaded to think that the improvements in dentistry have been greater, or that they have been made more rapidly than has been the general progress of the age, it will be well to remember that teeth were filled when the printing press was but in infancy, and that steamboats and railroads and telegraphs came into use long after artificial teeth were worn by George Washington, the first President, of this then experimental Republic. Then days and nights of most uncomfortable and weary travel were con-consumed in an overland journey from New York to Boston. Now, we sup and attend evening amusements in New York; then in flying palaces of rare woods and costly upholstery we retire to beds of luxury; and in the morning with carefully made toilets and with a *resume* of the news, as a relish, we breakfast in Boston. Then, to have transported by land the amount of freight that is now carried from Boston to New York in a single train of cars, in a few hours, would have upset the labor and taxed the resources of all New England for months. Then, to have journeyed from Boston to where the city of Detroit now stands, would been to bid farewell to friends and to place one's self beyond religious instruction or the watchful attentions of New England sheriffs. To-day, Chicago and even the Rocky Mountains, are but convenient places for replenishing the lunch basket or larder of the thousand who every year, over mountains, through valleys and beside rivers, in Pullman's winged hotels, go rushing across a continent, composedly viewing prairie and lake, forrest, fretting brooks, sierra and canyon and the silvered course of great rivers, and taking in, now and then a glimpse of a herd of wild buffalo, or camp and dance of wild aborigines. Hamlets and cities, the growth of a few months, are left behind in rapid succession, as on and on they rush to or from the golden State, the Atlantic or the the Pacific Coast.

Could the wisest statesman who lived at the commencement of the present century, be set down to-day in that great city of the West, once burned and twice built within the last forty years, and witness the passengers and freight passing East and West over the great trunk roads centering there, he would surely think that this was not the world in which he once lived, and that the inhabitants of this strange land were changing ends with the continent, so altered and improved have all things become, so different the bulk and modes of travel and business.

Take whatever example we please be it in agriculture, the arts or sciences, education, civil or religious freedom, the arts of war or those for promoting intercourse and preserving peace and thrift, the progress of the last seventy-five years has been greater than of centuries before.

And only abreast with the advance of the age, have marched the improvements in the science and practice of dentistry, the surgical or operative department of which when rightly understood and practiced, has become a legitimate branch of medicine, requiring the same general knowledge of medical science, as does the practice of aural, or general surgery, or ophthalmology.

The same general laws of health and disease, pervade alike the most vital and the most remote and unimportant organs of the human body. To comprehend the laws that govern either the physiological or pathological condition of any particular organ or member of the body, how to preserve health or intelligently treat any particular disease, be it either general or special, requires such a broad and special, knowledge of the laws of health, and the nature and treatment of disease, as would qualify the special practitioner to diagnose and acceptably treat the ordinary forms of disease met with by the family physician. Special knowledge of and unusual skill in the treatment of any particular form of disease, requires a general knowledge of the laws of formation, of growth, of the nature and results of different diseases, and of the treatment compatible with nature that is best calculated to restore diseased parts to healthy action and conditions. Treatment upon the human organism, either general or special, resting upon any other foundation than this is simply empirical.

We have heard much these many years about dentistry being a specialty in medcine, and that it ought to be so accredited by medical men; but if these views are correct to what extent should dentistry, as practiced at the present day, be so regarded? And to what extent should dentists, as a class, rank with surgeons, ophthalmologists and aurists as legitimate specialists in medical practice?

In inquiring into this question, it will be proper to allude briefly to the past history, and the present condition and needs of the profesion.

While history informs us that, as early as the days of Herodotus, dentistry was practiced, it is safe to say, that until a camparatively recent date, dental work of any kind, was exceedingly rare, and the business was not followed as a distinct calling. The little that was done was confined mainly to the carving and setting of artificial teeth from bone and ivory, possessing but little artistic taste or use. I make no new statement when I say that up to about the beginning of the present century, teeth were extracted by barbers and surgeons and that artificial teeth were carved and set by jewelers and silversmiths. Although teeth were then occasionally filled, the construction of a set of artificial teeth was regarded as the *ne plus ultra* of dental skill. At the same time this was looked upon simply as evidence of that peculiar mechanical ingenuity which enabled the artizan to turn his hand to an odd job outside of the regular routine of his trade. Dentistry was then merely mechanical; comparatively nothing being known either of dental histology, physiology, pathology or therapeutics.

Experience having demonstrated that fillings, though imperfect, would arrest decay in teeth, the demand for these operations increased; but up to this time they seemed to be empirical, the science of their beneficial effects not being understood. But as the period of the higher mission of dentistry—the saving of natural teeth—approached, Hunter, Blake, Fox and other medical men began to study more closely their structure, their physiological and pathological conditions, and their relations to the general system. Then followed the therapeutics of filling, and the science becoming better understood, a demand for better and more frequent operations upon the natural teeth arose, and for the purpose of making artificial teeth and the treatment of natural ones, a co-partnership between medical science and a mechanical art was entered into and conducted under the firm name of "dentistry".

Under this new condition of things, dentistry began to have a literature, and its practice was espoused by men of higher culture, to whom is offered a field for the exercise of more varied talents as well as one of greater usefulness. In a few years such men as Greenwood, Hudson, Hayden, Parmly, Flagg, Harris, Townsend, Clute, Westcott and Badger were found engaged in practice. The science and practice of dentistry, in its various departments, were pushed forward with great rapidity, and schools were formed, in which was given such medical and mechanical instruction as was thought necessary to qualify the student for practice in accordance with the standard sought to be established by such men as I have named, and with a view to making it a specialty in medicine.

Dentistry, thus given over to a special class of practitioners, has conquered its way, by its inherent importance to the welfare of man, up to the high position—taking its ablest representatives for the criterion—it now occupies, and in this growth it has approached nearer and nearer to its normal status and true mission, an integral part of the science and art of medicine.

In bringing about this result, whereby our practice has been settled upon a more scientific and practical basis, and a wider range and better class of operations brought into vogue our dental colleges have been greatly instrumental. The complaint, however is frequently made, and not altogether without grounds, that the standard actual, for graduation in these colleges is much below their standard as formulated; and that diplomas have in many instances, been conferred upon persons unworthy to recieve them. Still I feel that it would be unjust to say that the actual standard has not been fully up to the demand, either of the profession or the public. In fact, I believe our schools have been more anxious to graduate first-class practitioners than a large majority of the public have been willing to encourage and pay for first-class skill.

I am aware that it is said the demand of the age is for better dentistry. I do not deny that there is *need* of a better class of dentists, but at the same time, I believe that the average skill of the graduates of our colleges is really up to the demand of the general public. Let it not be inferred, by these remarks, that I wish, or intend to sanction, or apologise for the shortcomings of our colleges, in graduating those whom they know to be unfitted for practice and unworthy of the honor conferred upon them. I merely state what I regard to be a fact; they supply, not the *need*, but the actual demand of the public, in the productions which they send forth. The principal is not unlike the inexorable laws of supply and demand in trade. The people usually get what they appreciate and demand, whether it be in commerce, manufacture, education, or professional services. Let those, therefore, who desire to see our schools more exacting, as to the qualifications of their graduates, see to it, that not only by a correct example in practice on their own part, but also by a systematic, and correct course, of popular dental instruction, in public prints, journals and otherwise, the people are taught the importance of saving their natural teeth, and the difference between correct practice and quackery. Then, too, it will be well, for some of those, who find so much fault with our dental colleges, to take care that there be such an elimination of students, that those only, who have a sufficient amount of brains, and other requisite qualifications, to make good practitioners, be encouraged to enter the profession, and that such private instruction be imparted to them as shall qualify them to receive the greatest benefit from a high grade of teaching in our colleges, or else cease their fault-finding.

I do not wish to exonerate our schools from the just blame that should be attached to them; but, let it be remembered, that the sin of omission, on the part of the profession, is quite as great as the sin of commission, on the part of our colleges. Let him that is without sin, cast the first stone.

While no one can question that amongst the educated and better classes of people, there is an increasing demand for higher skill in saving natural teeth, no careful observer can have failed to notice the fact, that, within the last fifteen years, there has been an increasing demand for cheap sets of artificial teeth. The result of this is that the better class of men entering the profession are devoting their time, almost exclusively, to operative dentistry, in the service of patients who really appreciate skill: whilst the inferior men are turning their attention to low-priced, mechanical dentistry, and to inferior operations in filling teeth for such people as neither appreciate, nor are willing to pay for saving operations on the natural teeth.

That mechanical dentistry should have very largely fallen into the hands of this inferior class of practitioners will hardly be wondered at by those who have watched the history of that branch of practice. Up to about twenty years ago the mechanical department of the practice required a practical knowledge of selecting and compounding the materials out of which teeth were made; the hand and the eye of an artist were requisite to give them form and color; the management of heat in baking them; a knowledge of the nature of the precious metals and skill in working them, and a high order of mechanical talent in applying intricate mechanical laws, in fitting and rendering useful the different forms of plates, together with mechanical and artistic skill in adjusting the substitutes as to subserve the purposes for which they were intended. Since then, the manufacture of artificial teeth has become a distinct business, and they are now simply articles of commerce, bought by the piece, set or thousand; and to such perfection has this branch of manufacture been carried, that no dentist now thinks of making the teeth he uses. Plates of precious metal, requiring mechanical skill of a high order to manipulate, have, in a large majority of cases, been substituted by plates cast from baser metals, or by rubber vulcanized in molds, these requiring neither a high degree of judgment nor mechanical skill to accomplish results tolerably well, limited by the properties of the material used.

As a consequence, therefore, of these conditions, the surgical branch of dentistry, which, when practiced by competent men, allies it to medical science, has been constantly on the advance while that which is devoted to the setting of artificial teeth, has, in the last few years, been steadily retrograding and becoming more and more a trade. And so simple are the modes of attaining tolerable mechanical results, with the methods now usually employed in this department, that a high order of appropriate talent is, at the present time seldom, found devoting much time to it. By this I would not be understood as saying that this latter department does not need improving; for, when viewed as an art, he who has but moderate ideas of symmetry or harmony of expression and color is constantly pained by a lack of that artistic selection and arrangement of artificial teeth, which serves to restore to the face the shape and expression left upon it by the Creator—the absence of which, in artificial dentures, stamps him who should be an artist, an *artisan*—a mere mechanic—a libeller of the soul—a deformer of the human face, Divine.

At the present time there are abont 12,000 practicing dentists in the United States, about 2,000 of whom are regular or honorary graduates of either dental or medical schools. To offset the unworthy graduates by those practitioners, not graduates, who, by study and exertion have earned a deserved reputation and position in practice, would, I think, form a fair estimate of the really competent practitioners of scientific dentistry in the United States at the present day; making the number of the really qualified about one-sixth of the entire number. Assuming, therefore, that the one-sixth, as are the members of your Academy, sufficiently advanced in the knowledge of medical science to entitle them to the right to be regarded as special practitioners of medicine, it can hardly be expected that dentistry, when taken as a whole, would or ought to be so regarded by medical men.

This claim, while being justly made by some and freely acknowledged as to a portion of dental practitioners individually, has, not without cause, I think, been denied to the profession at large. The tastes, habits and acquirements of the two classes of dental practiioners are as divergent as are the characters of true science and mechanism; the practice of the one being established upon a medical basis, while the other relates only to a mechanical art. The practice of either branch, it is true, involves a limited knowledge of the other, but it is not necessary either for the surgical practitioner to be a practical mechanic, or the mechanician upon artificial work to understand the rationale of medical treatment, or to be an operator. In fact, the practice of both, by the same individual prevents the highest development of either, as would time spent in the manufacture of artificial legs by a surgeon, or the compounding, baking and coloring of artificial eyes by the ophthalmologist, serve but to retard the higher development of their specialties, or an attempt by the maker of limbs, eyes or optical instruments to practice general surgery, or the treatment of the eye, but degrade his own proper art.

As I have before stated, the yoking together of the two callings seemed to be a necessity of the then condition of the practice, at the time they were joined, and has resulted in great good. But the development of the practice has now brought us to a point where it is clear a new daparture should be taken, the, co-partnership dissolved, and each department followed as a distinct and separate calling, both no longer, either in private offices, or in our colleges, be taught as *one*; and the term "dentist" dropped from our nomenclature.

The true mission of medical science is to preserve or restore health and save life and limb, and not to make or have to do with the making of artificial substitutes any further than as they shall be made directly useful in subserving these purposes. Wig-making, and the manufacture of artificial limbs and eyes, are useful and respectable callings, and, when properly pursued, require a good degree not only of mechanical skill, but also of artistic taste; and as well almost might the making of these be taught in the medical college, as the making of sets of artificial teeth form part of the curriculum in a medical specialty. The long association of operative with mechanical dentistry, will make it somewhat more difficult, at first, to disconnect them in the minds of the public, than in practice, as separate callings; but no professional act would be so directly instrumental, in accomplishing this result, as to drop mechanical dentistry from the curriculum of our colleges, and employ the time usually devoted to the teaching and practical work in the manufacturing of artificial teeth, (a knowledge of and skill in which, is of no practical use in private practice, at the present day) to metallurgy, to mounting artificial teeth and to other laboratory work, to giving broader and more comprehensive instruction in the science of medicine in these schools, or else to incorporate them with the regular colleges of medicine, by the establishment of appropriate chairs and infirmaries for clinical teaching.

Let dental mechanics be otherwise taught, as a high mechanical art, and the calling fixed in the mind of the public as such, and, in a few years, the patient would as soon go to a maker of artificial legs for advice or treatment, in conservative surgery, or regarding amputation, as to the *dentician* or *dentificer*, for advice or services in the saving of his natural teeth, or their extraction.

To drop the teaching of mechanical dentistry in private offices and in our colleges, would, in a few years, permanently divide the practice, and very soon each town of any considerable size, would have one or more of these practitioners who, by relying upon success in this calling for support, and becoming personally responsible for what they did, would seek to redeem and elevate this particular art to the highest degree attainable; thereby enhancing the respectability and usefulness of their calling. And the *dentologist* would, by the broad and comprehensive teachings of medicine, become more thoroughly grounded in its science and be better qualified to take his rank with the other medically recognized specialists. With this thorough ground work laid, he would not only be better prepared to treat from a medical standpoint the diseases belonging to his province, but also to grapple successfully, by general treatment, with those hidden and hitherto ill-understood influences which serve to prevent perfect dental development, and also to counteract those pathological conditions which act as causes of disease in the teeth and tend to break down their tissues.

With the development of this higher mission of our profession there will be no occasion for the spectacle of dentology, with the grimace and shuffle of the mendicant, approaching the gates of the medical profession, and with downcast eyes begging a crumb of recognition. But with the accomplished separation of the two callings, heretofore combined in our practice, dentology, enriched by the experience and the special literature of the last half century, and the foundation of its practice laid exclusively in the science of medicine, rather than divided between that and a trade, the incongruity of the past will, in a few years, disappear, and by deriving its nourishment from the body of which it is a branch, it will become more and still more assimilated to the science and practice of medicine, and without demand, there will, both by the public and the medical faculty, be accorded, not to individual practitioners, but to the branch a full and cordial recognition as a specialty in medicine, which will attract more generally to its ranks, as to an agreeable and useful field of labor, men of earnestness, ability and culture, the peers of any in an honorable profession.

